# Relay-Assisted Communications over Multi-Cluster Two-Wave Fading Channels

**DOI:** 10.3390/s26051702

**Published:** 2026-03-08

**Authors:** Muhammad Junaid Rabbani, Zakir Hussain, Haider Mehdi, Shahzad Ashraf, Syed Muhammad Atif Saleem

**Affiliations:** 1 Department of Electrical Engineering, National University of Computer and Emerging Sciences, Karachi 75030, Pakistan; junaid.rabbani@nu.edu.pk (M.J.R.);; 2 Department of Computer Engineering, Gachon University, Seongnam 13120, Republic of Korea

**Keywords:** CCI, strictly positive secrecy capacity, MTW, THz, secrecy outage, intercept probability

## Abstract

This paper examines the secrecy performance of a decode-and-forward (DF) relay-assisted device-to-device (D2D) communication system operating over Terahertz (THz) channels in multi-cluster two-wave (MTW) fading environments. Eavesdroppers are located near the relay and the receiver, intercepting their respective signals. Co-channel interference (CCI) affecting the relay, receiver, and eavesdroppers is also considered. To counter fading, both the relay and the receiver employ Maximal Ratio Combining (MRC). The analysis uses a characteristic function (CF)-based approach to derive key secrecy metrics, such as secrecy outage probability, secrecy success probability, the probability of strictly positive secrecy capacity, and intercept probability. The derived expressions are dependent on the characteristics of the THz, MTW fading, and CCI parameters. Finally, the system’s performance is then evaluated numerically for a range of channel and interference parameters.

## 1. Introduction

Device-to-device (D2D) communication is a device-centric method that generally operates without a direct connection to the network infrastructure [1,2,3]. For extended ranges, relay-based D2D is often employed. In decode-and-forward (DF) communication systems, for instance, an intermediate device decodes and subsequently forwards the signal to its receiver [4]. A prevalent issue in wireless systems is co-channel interference (CCI), which arises when multiple devices utilize the same channel resources [5]. This research also considers the specific characteristics of Terahertz (THz) channels [6]. The inherent weakness of wireless communication is the possibility of signal interception. A transmitted signal can be intercepted by any unauthorized party (an eavesdropper) within its range, rather than only the intended receiver. Mathematically, physical layer security (PLS) analysis is employed to quantify this level of security [7].

The literature review and background work can be summarized as follows: The authors of [8] analyzed a PLS framework that integrates intelligent reflecting surfaces (IRSs), non-orthogonal multiple access (NOMA), and cooperative jamming to enhance spectral efficiency and secure communication. They contribute a theoretical performance analysis by deriving an expression of the ergodic secrecy rate (ESR) to evaluate the system under Rayleigh fading. In [9], the authors theoretically prove that traditional far-field multiple-antenna techniques and Frequency Diverse Arrays (FDAs) are ineffective in providing physical-layer range security in the Terahertz band. To overcome this limitation and optimize the secrecy rate, they propose a near-field Widely Spaced Array (WSA) architecture combined with hybrid beamforming design and a Non-Constrained Optimum Approaching (NCOA) algorithm. Meanwhile, [10] investigates a secure low Earth orbit (LEO) satellite communication framework that integrates reconfigurable intelligent surface (RIS) with THz technology to mitigate atmospheric scintillation and pointing errors. They derive analytical expressions for secrecy outage probability and average secrecy rate. In [11], the authors established a unified analytical framework for investigating PLS over mixture Gamma distributed fading channels with discrete inputs. They derive closed-form expressions for the average secrecy rate and secrecy outage probability. In [12], the authors address the limitations of conventional fading models in capturing mmWave signal fluctuations. This study conducts a comprehensive PLS analysis using the Fluctuating Two-Ray (FTR) model. The authors derive the exact analytical expressions for secrecy capacity, secrecy outage probability, and the probability of strictly positive secrecy capacity. In [13], authors analyze PLS over M-distributed fading channels, deriving the exact integral expressions for secrecy outage probability and ergodic secrecy capacity. They arrive at closed-form lower bounds for secrecy outage probability and ergodic secrecy capacity to reduce computational complexity. They establish a closed-form exact expression for the probability of strictly positive secrecy capacity. In the next paragraph, the key contributions of this work are given.

This work analyzes the PLS of DF-based D2D systems operating over THz channels. However, unlike prior research, the presence of CCI is also considered. We assume the CCI originates from uncoordinated external devices. Both the D2D and the interference links are modeled using the novel multi-cluster two-wave (MTW) distribution proposed in [14]. MTW is a unified framework that encompasses both two-wave with diffuse power (TWDP) and κ−μ fading conditions. Maximal Ratio Combining (MRC) is deployed at both the relay and the receiver to mitigate fading effects. By utilizing a characteristic function (CF) approach, we derive analytical expressions for the secrecy outage probability, secrecy success probability, the probability of strictly positive secrecy capacity, and intercept probability. To the best of our knowledge, this is the first work to investigate the secrecy performance of DF/MRC-based D2D systems under MTW fading and CCI constraints in the THz band. A comparison of our key contributions with previous works is summarized in Table 1.

The work is presented as follows: in Section 2, system model and mathematical derivations are given; in Section 3, numerical results are analyzed; and in Section 4, this work is concluded.

## 2. Problem Description and Formulation

**Figure 1:**
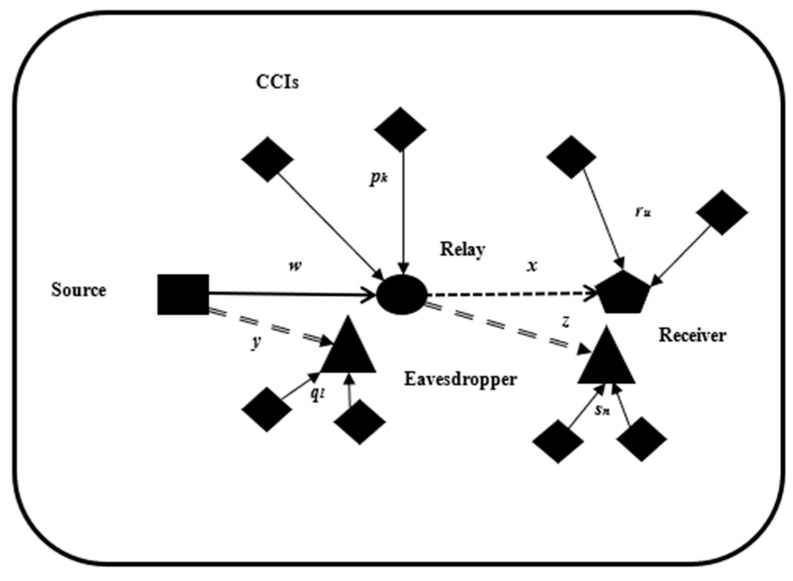
System model.

A D2D network with DF relaying over a novel MTW fading channel is shown in Figure 1. The network parameters are outlined in Table 2. In this environment, independent and non-identically distributed co-channel interferers impact the D2D network. The relay, the relay’s eavesdropper, the receiver, and the receiver’s eavesdropper are subjected to K, L, U and N interferers, respectively. Noise is neglected in this analysis. As the system is considered interference-limited, the interference power renders the effects of noise negligible.

Secrecy outage probability (SOP) is considered here to evaluate the secrecy performance of a D2D network. It is defined as the probability that the secrecy capacity (SC) is below a target capacity, $C_{th}$. The SC is given as [7]:

(1) $$SC=min\left[{\left\{\frac{1}{2}\log_{2}\left(1+\gamma_{SR}\right)-\frac{1}{2}\log_{2}\left(1+\gamma_{RE1}\right)\right\}}^{+},{\left\{\frac{1}{2}\log_{2}\left(1+\gamma_{RD}\right)-\frac{1}{2}\log_{2}\left(1+\gamma_{DE2}\right)\right\}}^{+}\right]$$

where min(,) is the minimum of two, ${\left\{t\right\}}^{+}=max\{t,0\}$, $\gamma_{SR}$ and $\gamma_{RD}$ are signal-to-interference ratios (SIRs) at relay and D2D receiver, respectively. Also, $\gamma_{RE1}$ and $\gamma_{DE2}$ are SIRs at eavesdropper near relay and eavesdropper near D2D receiver, respectively. Hence, SOP is given as:

(2) $$P_{SOP}=\mathrm{Pr}\left\{\mathrm{SC}<C_{th}\right\}$$

(3) $$P_{SOP}=\mathrm{Pr}\left\{\gamma_{SR}<2^{2C_{th}}\gamma_{RE1}+2^{2C_{th}}-1\right\}+\mathrm{Pr}\left\{\gamma_{RD}<2^{2C_{th}}\gamma_{DE2}+2^{2C_{th}}-1\right\}\;\;-\mathrm{Pr}\left\{\gamma_{SR}<2^{2C_{th}}\gamma_{RE1}+2^{2C_{th}}-1\right\}\mathrm{Pr}\left\{\gamma_{RD}<2^{2C_{th}}\gamma_{DE2}+2^{2C_{th}}-1\right\}$$

The signal-to-interference ratio (SIR) at relay incorporating M branch diversity is:

(4) $$\frac{S_{R}}{S_{I}}=\frac{P_{S}\sum_{m=1}^{M}h_{R}\alpha_{m}}{\sum_{k=1}^{K}P_{R,k}\beta_{R,k}h_{R,k}}$$

where D2D signal power is *S_R_*, CCI power is *S_I_*, the D2D source transmitted power is *P_S_* and the source-to-relay THz channel parameter is $h_{R}={\left(h_{pR}h_{aR}h_{mR}\right)}^{2}$ [6]. $\alpha_{m}$ is the MTW channel gain of D2D signal in the m−th branch. $P_{R,k}$ is the power, the THz channel gain is $h_{R,k}={\left(h_{pR,k}h_{aR,k}h_{mR,k}\right)}^{2}$ and $\beta_{R,k}$ is the MTW gain for the k−th CCI in the m−th branch. The path loss is $h_{pR}=\frac{c\sqrt{G_{TX,S}G_{RX,R,m}}}{4\pi wf}$, where carrier frequency is *f* and speed of light is *c*. Transmitter and receiver antenna gains in the m−th diversity branch at relay are $G_{TX,S}$ and $G_{RX,R,m}$, respectively. Here, it is considered that all $G_{RX,R,m}$ are equal. The parameter $h_{aR}=e^{-\frac{1}{2}k_{aR}\left(f\right)w}$ is the molecular absorption loss. Absorption coefficient is $k_{aR}(f)$.

$k_{aR}(f)\;=\;g_{R}(f)\;+\;y_{1R}(f,\;\nu )\;+\;y_{2R}(f,\;\nu )$, where $g_{R}(f)\;=\;c_{0R}\;+\;c_{1R}f\;+\;c_{2R}f^{2}\;+\;c_{3R}f^{3}$, $y_{1R}\left(f,v_{R}\right)=\frac{0.2205v_{R}\left(0.1303v_{R}+0.0294\right)}{{\left(0.4093v_{R}+0.0925\right)}^{2}+{\left(\frac{100f}{c}-10.835\right)}^{2}}$, $y_{2R}\left(f,v_{R}\right)=\frac{2.014v_{R}\left(0.1702v_{R}+0.0303\right)}{{\left(0.537v_{R}+0.0956\right)}^{2}+{\left(\frac{100f}{c}-12.664\right)}^{2}}$, $c_{0R}\;=\;(-6.36\;\times \;10^{-3})$, $c_{1R}\;=\;(9.06\;\times \;10^{-14}\;Hz^{-1})$, $c_{2R}\;=\;(-3.94\times 10^{-25}\;Hz^{-2})$, $c_{3R}\;=\;(5.54\times 10^{-37}\;Hz^{-3})$ and $v_{R}=\frac{\phi_{R}p_{wR}\left(T_{R},p_{R}\right)}{100p_{R}}$ is defined as the water vapor’s volume mixing ratio, where $\phi_{R}$ is defined as the relative humidity, $p_{R}$ gives the pressure and $p_{wR}(T_{R},p_{R})$ is defined as the saturated water vapor partial pressure. $p_{wR}(T_{R},p_{R})$ is defined as:
  $$p_{wR}\left(T_{R},p_{R}\right)=q_{1R}\left(q_{2R}+q_{3R}p_{hR}\right)\exp\left(\frac{q_{4R}\left(T_{R}-q_{5R}\right)}{T_{R}-q_{6R}}\right)$$

Now, $q_{1R}\;=\;6.1121$, $q_{2R}\;=\;1.0007$, $q_{3R}\;=\;3.46\;\times \;10^{-6}\;hPa^{-1}$, $q_{4R}\;=\;17.502$, q5R=273.15 K∘ and q6R=32.18 K∘. Also, the pressure $p_{hR}$ is in hectopascal and the absolute temperature is $T_{R}$. $h_{mR}\approx A\exp\left(\frac{-2r^{2}}{\omega_{eq}^{2}}\right)$ is the misalignment due to the pointing error, $A={\left(\mathrm{erf}\left(u\right)\right)}^{2}$, erf() gives the error function, $u=\frac{a\sqrt{\pi }}{\omega_{x}\sqrt{2}}$, *a* is detection area radius of the receiver, $\omega_{x}$ is transmitter beam waist and $\omega_{eq}^{2}=\frac{\omega_{x}^{2}\sqrt{\pi }\mathrm{erf}\left(u\right)}{2u\exp\left(-u^{2}\right)}$ gives the equivalent beam width. The parameter r is the pointing error; it is defined as the radial distance between the reception area and the transmitter beam focus centers. Pointing errors are same for all branches. Now, $h_{pR,k}=\frac{c\sqrt{G_{TX,k}G_{RX,R,m}}}{4\pi p_{k}f}$ where $G_{TX,k}$ is the transmit antenna gain of the k−th CCI, $h_{aR,k}=e^{-\frac{1}{2}k_{aR}\left(f\right)p_{k}}$ and $h_{mR,k}\approx A\exp\left(\frac{-2r_{k}^{2}}{\omega_{eq}^{2}}\right)$ where $r_{k}$ is the pointing error of the k−th CCI. No diversity conditions are assumed at the eavesdropper near relay. The SIR at the eavesdropper near relay is:

(5) $$\gamma_{RE1}=\frac{S_{E1}}{S_{E1I}}=\frac{P_{S}h_{E1}\Omega_{E1}}{\sum_{l=1}^{L}P_{E1,l}\Omega_{E1,l}h_{E1I,l}}$$

where $S_{E1}$ is the signal power at eavesdropper, $S_{E1I}$ is the CCI power at eavesdropper and $h_{E1}={\left(h_{pR,E1}h_{aR,E1}h_{mR,E1}\right)}^{2}$ defines the THz channel gain of the source-to-eavesdropper channel. $\Omega_{E1}=E\left(\alpha_{E1}\right)$ where $\alpha_{E1}$ is MTW gain for the source-to-eavesdropper channel and E(.) is the expectation operator. In $h_{E1},$ $h_{pR,E1}=\frac{c\sqrt{G_{TX,S}G_{RX,R,E1}}}{4\pi yf}$ where $G_{RX,R,E1}$ is the eavesdropper receive antenna gain. The parameter $h_{aR,E1}=e^{-\frac{1}{2}k_{aR}\left(f\right)y}$ and $h_{mR,E1}\approx A\exp\left(\frac{-2r_{E1}^{2}}{\omega_{eq}^{2}}\right)$ where $r_{E1}$ is the eavesdropper’s pointing error. Also, l−th CCI power at eavesdropper is $P_{E1,l}$, $\Omega_{E1,l}=E\left(\beta_{E1,l}\right)$ where $\beta_{E1,l}$ is the l−th CCI MTW gain at eavesdropper and $h_{E1I,l}={\left(h_{pR,E1,l}h_{aR,E1,l}h_{mR,E1,l}\right)}^{2}$ is l−th CCI THz channel gain at eavesdropper. $h_{pR,E1,l}=\frac{c\sqrt{G_{TX,E1,l}G_{RX,R,E1}}}{4\pi q_{l}f}$ where $G_{TX,E1,l}$ is the transmitter antenna gain of the l−th CCI, $h_{aR,E1,l}=e^{-\frac{1}{2}k_{aR}\left(f\right)q_{l}}$ and $h_{mR,E1,l}\approx A\exp\left(\frac{-2r_{E1,l}^{2}}{\omega_{eq}^{2}}\right)$ where $r_{E1,l}$ is the pointing error of the l−th CCI. From (3), by considering threshold $R_{1}=2^{2C_{th}}\gamma_{RE1}+2^{2C_{th}}-1$,

(6) $$P_{1}=\mathrm{Pr}\left\{\gamma_{SR}<2^{2C_{th}}\gamma_{RE1}+2^{2C_{th}}-1\right\}=\mathrm{Pr}\left\{\frac{S_{R}}{S_{I}}<R_{1}\right\}=\mathrm{Pr}\left\{R_{1}S_{I}>S_{R}\right\}$$

The outage probability $P_{1}$ is defined as the probability of SIR of the source-to-relay channel falling below a defined threshold value $R_{1}$, i.e., $\mathrm{Pr}\left\{R_{1}S_{I}>S_{R}\right\}$. The decision variable, $\theta_{1}=R_{1}S_{I}-S_{R}$, CF is [12]:

$$\begin{array}{c} \phi_{1}\left(\omega \right)=E\left(e^{j\omega \left(RS_{I}-S_{R}\right)}\right)\\ =E\left(e^{j\omega RS_{I}}e^{j\omega \left(-S_{R}\right)}\right)\\ =E\left(e^{j\omega R\left[\sum_{k=1}^{K}P_{R,k}\beta_{R,k}h_{R,k}\right]}e^{-j\omega P_{S}\sum_{m=1}^{M}h_{R}\alpha_{m}}\right)\\ =\prod_{m=1}^{M}E\left(e^{-j\omega P_{S}\alpha_{m}h_{R}}\right)\prod_{k=1}^{K}E\left(e^{j\omega RP_{R,k}\beta_{R,k}h_{R,k}}\right) \end{array}$$

(7) $$\begin{array}{c} \phi_{1}\left(\omega \right)=\prod_{m=1}^{M}\left\{{\left\{\frac{\mu_{m}\left(1+\lambda_{m}\right)}{\mu_{m}\left(1+\lambda_{m}\right)+j\omega P_{S}\Omega_{m}h_{R}}\right\}}^{\mu_{m}}\exp\left\{\frac{-j\omega P_{S}\Omega_{m}h_{R}\mu_{m}\lambda_{m}}{\mu_{m}\left(1+\lambda_{m}\right)+j\omega P_{S}\Omega_{m}h_{R}}\right\}\right.\\ \left.\times \prod_{i_{m}=1}^{I_{m}}I_{0}\left(\frac{-j\omega P_{S}\Omega_{m}h_{R}\mu_{m}\lambda_{m}\Delta_{i_{m}}}{\mu_{m}\left(1+\lambda_{m}\right)+j\omega P_{S}\Omega_{m}h_{R}}\right)\right\}\times \prod_{k=1}^{K}\left\{{\left\{\frac{\mu_{R,k}\left(1+\lambda_{R,k}\right)}{\mu_{R,k}\left(1+\lambda_{R,k}\right)-j\omega R_{1}P_{R,k}\Omega_{R,k}h_{R,k}}\right\}}^{\mu_{R,k}}\right.\\ \left.\times \exp\left\{\frac{j\omega P_{R,k}\Omega_{R,k}h_{R,k}\mu_{R,k}\lambda_{R,k}}{\mu_{R,k}\left(1+\lambda_{R,k}\right)-j\omega R_{1}P_{R,k}\Omega_{R,k}h_{R,k}}\right\}\times \prod_{i_{R,k}=1}^{I_{R,k}}I_{0}\left(\frac{j\omega P_{R,k}\Omega_{R,k}h_{R,k}\mu_{R,k}\lambda_{R,k}\Delta_{i_{R,k}}}{\mu_{R,k}\left(1+\lambda_{R,k}\right)-j\omega R_{1}P_{R,k}\Omega_{R,k}h_{R,k}}\right)\right\}. \end{array}$$

The D2D signal’s MTW parameters [12] in the m−th branch of relay are as follows: $\mu_{m}$ gives the number of clusters, $\lambda_{m}$ is defined as the ratio of the specular components’ powers to the diffuse components’ powers from all the clusters, $\Delta_{i_{m}}$ describes the asymmetry between the specular components of the cluster, $0\leq \sum_{i_{m}=1}^{\mu_{m}}\Delta_{i_{m}}\leq 1$, $0\leq I_{m}\leq \mu_{m}$ where $I_{m}$ is the number of clusters containing two specular components and $\Omega_{m}=E\left[\alpha_{m}\right]$ gives the average power. The modified Bessel function of the first kind is $I_{0}\left(.\right)$. Similarly, $\mu_{R,k}$, $\lambda_{R,k}$, $\Delta_{i_{R,k}}$ with $0\leq \sum_{i_{R,k}=1}^{\mu_{R,k}}\Delta_{i_{R,k}}\leq 1$ and $I_{R,k}$ with $0\leq I_{R,k}\leq \mu_{R,k}$ are MTW parameters of the k−th CCI at the relay. These are defined in a similar manner as the MTW parameters of the D2D signal. Also, $\Omega_{R,k}=E\left[\beta_{R,k}\right]$. The outage probability is given by:

(8) P1=12+1π∫0∞Imϕ1ωωdω

The integrals in (8) can be solved with the help of MATLAB R2016a built-in ‘integral’ function based on global adaptive quadrature. The convergence criteria were determined by the default error tolerances: a relative tolerance of $10^{-6}$ and an absolute tolerance of $10^{-10}$. Infinite integration limits were handled via the function’s internal variable transformation strategies. At the receiver, a Q-branch MRC scheme is deployed. The SIR is:

(9) $$\frac{S_{D}}{S_{I,D}}=\frac{P_{R}\sum_{q=1}^{Q}h_{D}\alpha_{D,q}}{\sum_{u=1}^{U}P_{D,u}\beta_{D,u}h_{D,u}}$$

where $S_{D}$ is the D2D power, CCI power is $S_{I,D}$, power transmitted from the relay is $P_{R}$, $\alpha_{D,q}$ is the MTW gain for the relay-to-receiver transmission channel in the q−th branch, $P_{D,u}$ is the u−th CCI power and the u−th CCI MTW gain is $\beta_{D,u}$. $h_{D}={\left(h_{pD}h_{aD}h_{mD}\right)}^{2}$,$h_{pD}=\frac{c\sqrt{G_{TX,R}G_{RX,D,q}}}{4\pi xf}$ where $G_{TX,R}$ is the transmitter antenna gain and $G_{RX,D,q}$ is the receiver antenna gain in the q−th branch, respectively. $h_{aD}=e^{-\frac{1}{2}k_{aR}\left(f\right)x}$ and $h_{mD}\approx A\exp\left(\frac{-2r_{D}^{2}}{\omega_{eq}^{2}}\right)$; in $h_{mD},$ the pointing error is $r_{D}$. Pointing errors are same for all branches. Furthermore, $h_{D,u}={\left(h_{pD,u}h_{aD,u}h_{mD,u}\right)}^{2}$, $h_{pD,u}=\frac{c\sqrt{G_{TX,u}G_{RX,D,q}}}{4\pi r_{u}f}$ where $G_{TX,u}$ is the transmitter antenna gain of the u−th CCI,$\;h_{aD,u}=e^{-\frac{1}{2}k_{aR}\left(f\right)r_{u}}$ and $h_{mD,u}\approx A\exp\left(\frac{-2r_{D,u}^{2}}{\omega_{eq}^{2}}\right)$ where $r_{D,u}$ is the u−th CCI’s pointing error. It is assumed that all $G_{RX,D,q}$ are equal. No diversity conditions are assumed at the eavesdropper near receiver. The SIR at the eavesdropper near receiver is:

(10) $$\gamma_{DE2}=\frac{S_{E2}}{S_{E2I}}=\frac{P_{R}h_{E2}\Omega_{E2}}{\sum_{n=1}^{N}P_{E2,n}\Omega_{E2,n}h_{E2I,n}}$$

where $S_{E2}$ is the signal power, CCI power is $S_{E2I}$ and the THz channel gain for the relay-to-eavesdropper channel is $h_{E2}={\left(h_{pD,E2}h_{aD,E2}h_{mD,E2}\right)}^{2}$. $\Omega_{E2}=E\left(\alpha_{E2}\right)$ where $\alpha_{E2}$ is the MTW gain of the relay-to-eavesdropper channel. $h_{pD,E2}=\frac{c\sqrt{G_{TX,R}G_{RX,D,E2}}}{4\pi zf}$ where $G_{RX,D,E2}$ is the eavesdropper receive antenna gain. The parameter $h_{aD,E2}=e^{-\frac{1}{2}k_{aR}\left(f\right)z}$ and $h_{mD,E2}\approx A\exp\left(\frac{-2r_{E2}^{2}}{\omega_{eq}^{2}}\right)$ where $r_{E2}$ is the eavesdropper’s pointing error. $P_{E2,n}$ is the power of the n−th CCI at the eavesdropper, $\Omega_{E2,n}=E\left(\beta_{E2,n}\right)$ where $\beta_{E2,n}$ is the n−th CCI MTW gain at the eavesdropper and $h_{E2I,n}={\left(h_{pD,E2,n}h_{aD,E2,n}h_{mD,E2,n}\right)}^{2}$ is the THz channel parameter of the n−th CCI at the eavesdropper. $h_{pD,E2,n}=\frac{c\sqrt{G_{TX,E2,n}G_{RX,D,E2}}}{4\pi s_{n}f}$ where $G_{TX,E2,n}$ is the n−th CCI’s transmit antenna gain, $h_{aD,E2,n}=e^{-\frac{1}{2}k_{aR}\left(f\right)s_{n}}$ and $h_{mD,E2,n}\approx A\exp\left(\frac{-2r_{E2,n}^{2}}{\omega_{eq}^{2}}\right)$, where $r_{E2,n}$ is the n−th CCI’s pointing error. From (3), by considering threshold $R_{2}=2^{2C_{th}}\gamma_{DE2}+2^{2C_{th}}-1$,

(11) $$P_{2}=\mathrm{Pr}\left\{\gamma_{RD}<2^{2C_{th}}\gamma_{DE2}+2^{2C_{th}}-1\right\}=\mathrm{Pr}\left\{\frac{S_{D}}{S_{I,D}}<R_{2}\right\}=\mathrm{Pr}\left\{R_{2}S_{I,D}>S_{D}\right\}$$

For relay-to-receiver channel, outage probability is defined as $P_{2}=\mathrm{Pr}\left(RS_{I,D}>S_{D}\right)$. The decision parameter, $\theta_{D}=RS_{I,D}-S_{D}$, CF is:

(12) $$\phi_{2}\left(\omega \right)=\prod_{q=1}^{Q}\left\{{\left\{\frac{\mu_{D,q}\left(1+\lambda_{D,q}\right)}{\mu_{D,q}\left(1+\lambda_{D,q}\right)+j\omega P_{R}\Omega_{D,q}h_{D}}\right\}}^{\mu_{D,q}}\exp\left\{\frac{-j\omega P_{R}\Omega_{D,q}h_{D}\mu_{D,q}\lambda_{D,q}}{\mu_{D,q}\left(1+\lambda_{D,q}\right)+j\omega P_{R}\Omega_{D,q}h_{D}}\right\}\right.\;\;\left.\times \prod_{i_{D,q}=1}^{I_{D,q}}I_{0}\left(\frac{-j\omega P_{R}\Omega_{D,q}h_{D}\mu_{D,q}\lambda_{D,q}\Delta_{i_{D,q}}}{\mu_{D,q}\left(1+\lambda_{D,q}\right)+j\omega P_{R}\Omega_{D,q}h_{D}}\right)\right\}\times \prod_{u=1}^{U}\left\{{\left\{\frac{\mu_{D,u}\left(1+\lambda_{D,u}\right)}{\mu_{D,u}\left(1+\lambda_{D,u}\right)-j\omega R_{2}P_{D,u}\Omega_{D,u}h_{D,u}}\right\}}^{\mu_{D,u}}\right.\;\;\left.\;\times \exp\left\{\frac{j\omega P_{D,u}\Omega_{D,u}h_{D,u}\mu_{D,u}\lambda_{D,u}}{\mu_{D,u}\left(1+\lambda_{D,u}\right)-j\omega R_{2}P_{D,u}\Omega_{D,u}h_{D,u}}\right\}\times \prod_{i_{D,u}=1}^{I_{D,u}}I_{0}\left(\frac{j\omega P_{D,u}\Omega_{D,u}h_{D,u}\mu_{D,u}\lambda_{D,u}\Delta_{i_{D,u}}}{\mu_{D,u}\left(1+\lambda_{D,u}\right)-j\omega R_{2}P_{D,u}\Omega_{D,u}h_{D,u}}\right)\right\}.$$

where $\mu_{D,q}$, $\lambda_{D,q}$, $\Delta_{i_{D,q}}$,$\;I_{D,q}$ and $\Omega_{D,q}=E\left[\alpha_{D,q}\right]$ are the MTW distribution parameters of the D2D signal in the q−th branch. Now, $\Omega_{D,u}=E\left[\beta_{D,u}\right]$, $\mu_{D,u}$, $\lambda_{D,u}$, $\Delta_{i_{D,u}}$ and $I_{D,u}$ are the MTW parameters for the u−th interferer. Outage probability is given as:

(13) P2=12+1π∫0∞Imϕ2ωωdω.

Now, based on (3), (8) and (13), the SOP of the D2D system is:

(14) $$P_{SOP}=P_{1}+P_{2}-P_{1}P_{2}.$$

From (14), the overall secrecy success probability (SSP) condition for the D2D system is

(15) $$S_{S}=1-P_{SOP}.$$

The strictly positive secrecy capacity (SPSC) is an important benchmark. SPSC probability given as [7]

(16) $$P_{SPSC}=\mathrm{Pr}\left\{\mathrm{SC}>0\right\}.$$

$$P_{SPSC}=Pr\left\{\left(\frac{1}{2}\log_{2}\left(1+\gamma_{SR}\right)-\frac{1}{2}\log_{2}\left(1+\gamma_{RE1}\right)\right)>0\right\}\;\;\times \;Pr\left\{\left(\frac{1}{2}\log_{2}\left(1+\gamma_{RD}\right)-\frac{1}{2}\log_{2}\left(1+\gamma_{DE2}\right)\right)>0\right\}\;$$

(17) $$P_{SPSC}=\mathrm{Pr}\left\{\gamma_{SR}>\gamma_{RE1}\right\}\mathrm{Pr}\left\{\gamma_{RD}>\gamma_{DE2}\right\}$$

(18) $$P_{SPSC}=\left(1-P_{3}\right)\left(1-P_{4}\right)$$

where

(19) P3=12+1π∫0∞Imϕ1ωωdω,     with     R1=γRE1

and,

(20) P4=12+1π∫0∞Imϕ2ωωdω,     with     R2=γDE2.

Intercept probability (IP) is the probability of an eavesdropper succeeding in intercepting the desired signal. Such condition shows the probability that SC is less than zero [15]. The IPs at the relay and receiver are, respectively, as follows:

(21) $$P_{IPR}=Pr\left\{\left(\frac{1}{2}\log_{2}\left(1+\gamma_{SR}\right)-\frac{1}{2}\log_{2}\left(1+\gamma_{RE1}\right)\right)<0\right\}=Pr\left\{\gamma_{SR}<\gamma_{RE1}\right\}=P_{3}$$

and,

(22) $$P_{IPD}=Pr\left\{\left(\frac{1}{2}\log_{2}\left(1+\gamma_{RD}\right)-\frac{1}{2}\log_{2}\left(1+\gamma_{DE2}\right)\right)<0\right\}=Pr\left\{\gamma_{RD}<\gamma_{DE2}\right\}=P_{4}$$

In the subsequent special scenario, outage capacity probability (OCP), without any eavesdroppers in the D2D system, is presented. OCP is defined as the probability when D2D system’s capacity falls below a target capacity, $C_{th}$. Overall OCP of the D2D system is given as:

(23) $$P_{OCP}=\mathrm{Pr}\left\{\gamma_{SR}<2^{2C_{th}}-1\right\}+\mathrm{Pr}\left\{\gamma_{RD}<2^{2C_{th}}-1\right\}\;\;-\mathrm{Pr}\left\{\gamma_{SR}<2^{2C_{th}}-1\right\}\mathrm{Pr}\left\{\gamma_{RD}<2^{2C_{th}}-1\right\},$$

where

(24) P5=12+1π∫0∞Imϕ1ωωdω,    with    R1=22Cth−1

and,

(25) P6=12+1π∫0∞Imϕ2ωωdω,    with    R2=22Cth−1.

## 3. Results

Results are presented here by using the analytical expressions of Section 2, under various conditions of the channel and CCI. THz channel parameter values are based on [6]. In Table 3, fixed parameter values are shown.

**Figure 2:**
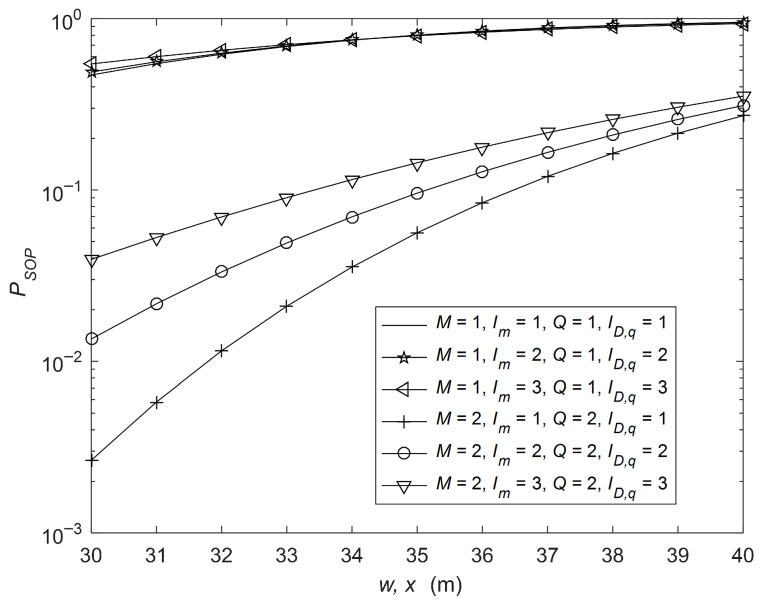
SOP analysis with various values of source-to-relay and relay-to-receiver distances.

**Figure 3:**
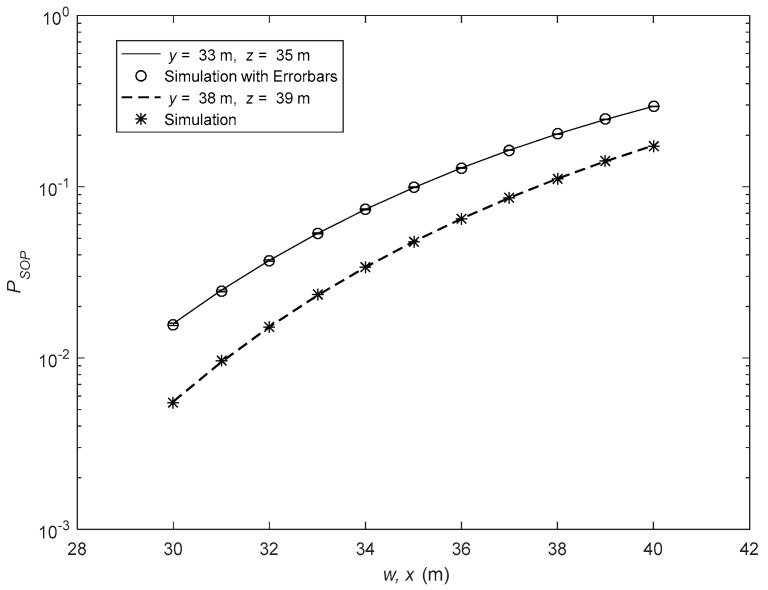
SOP performance with various values of source-to-eavesdropper and relay-to-eavesdropper distances.

In Figure 2, the SOP analysis is presented for various source-to-relay and relay-to-receiver distances. The parameters are as follows: $r_{E1}$ = 15 cm, $r_{E1,l}$ = [9, 8] cm, $\lambda_{m}$ = [20, 25], $\mu_{m}$ = [4, 3], $\Delta_{i_{m}}$ = [0.3, 0.4], $\mu_{R,k}$ = [2, 3], $\lambda_{R,k}$= [15, 10], $\Delta_{i_{R,k}}$ = [0.5, 0.4], $\mu_{D,q}$ = [3, 4], $\lambda_{D,q}$= [25, 20], $\Delta_{i_{D,q}}$ = [0.3, 0.2], $\mu_{D,u}$ = [3, 2], $\lambda_{D,u}$= [15, 20], $\Delta_{i_{D,u}}$ = [0.4, 0.6], $r_{E2}$ = 14 cm, $r_{E2,n}$ = [10, 8] cm, $I_{R,k}$ = 1 and $I_{D,u}$ = 1. It can be seen from the figure that by increasing source-to-relay and relay-to-receiver distances, SOP increases. Furthermore, under diversity conditions, SOP is decreased. Hence, for the following analysis, diversity conditions are considered, i.e., M = 2 and Q = 2. Also, as the values of $I_{m}$ and $I_{D,q}$ increase from 1 to 3, the SOP of the system increases. Hence, for the following analysis, $I_{m}$ and $I_{D,q}$ are fixed at 1. In Figure 3, the SOP performance is shown for various source-to-eavesdropper and relay-to-eavesdropper distances. The parameters are as follows: $r_{E1}$ = 15 cm, $r_{E1,l}$ = [9, 8] cm, $\lambda_{m}$= [20, 25], $\mu_{m}$ = [3, 4], $\Delta_{i_{m}}$ = [0.4, 0.2], $\mu_{R,k}$ = [1, 1], $\lambda_{R,k}$= [15, 10], $\Delta_{i_{R,k}}$ = [0.5, 0.4], $\mu_{D,q}$ = [4, 3], $\lambda_{D,q}$= [35, 30], $\Delta_{i_{D,q}}$ = [0.3, 0.5], $\mu_{D,u}$ = [1, 1], $\lambda_{D,u}$= [15, 10], $\Delta_{i_{D,u}}$ = [0.4, 0.6], $r_{E2}$ = 14 cm, $r_{E2,n}$ = [10, 8] cm, $I_{R,k}$ = 1 and $I_{D,u}$ = 1. It is obvious from the figure that, as the source-to-eavesdropper and relay-to-eavesdropper distances are increased, SOP is decreased. It is because, as the eavesdroppers move away from the D2D source and relay, the intercepted signal powers decrease at both eavesdroppers. Furthermore, it is also observed that the numerical analysis based on the expressions are in close agreement with the simulation results. Trial count for the simulations is 10^6^. Figure 3 also includes error bars representing 95% confidence intervals.

**Figure 4:**
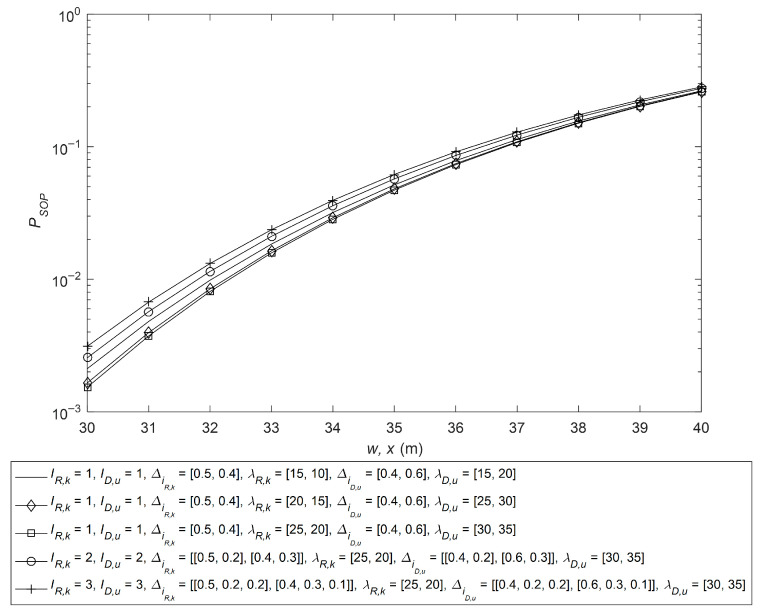
SOP analysis with various CCI MTW parameters at the relay and the receiver.

In Figure 4, SOP is given with different MTW CCI parameters at the relay and receiver. The parameters are as follows: $r_{E1}$ = 15 cm, $r_{E1,l}$ = [9, 8] cm, $\lambda_{m}$= [20, 25], $\mu_{m}$ = [4, 3], $\Delta_{i_{m}}$ = [0.3, 0.4], $\mu_{R,k}$ = [4, 4], $\mu_{D,q}$ = [3, 4], $\lambda_{D,q}$= [25, 20], $\Delta_{i_{D,q}}$ = [0.3, 0.2], $\mu_{D,u}$ = [3, 4], $r_{E2}$ = 14 cm and $r_{E2,n}$ = [10, 8] cm. It can be observed from the figure that, by varying the MTW parameters of CCI at the relay and receiver, slight variations in SOP are observed. Various CCI MTW parameters have a negligible effect on the SOP because the system’s performance is largely governed by the aggregate average power of interference. Consequently, variations in the fading parameters play a secondary role and do not significantly influence the SOP. For the rest of the analysis, the values of $I_{R,k}$ and $I_{D,u}$ are fixed at 1. Also, it is observed from the figure that maximum SOP deviation occurs at *w*, and *x* = 30 m. For the worst case, SOP is *P_SOP,W_* = 0.003123, and for the best case, SOP is *P_SOP,B_* = 0.001533. The maximum SOP deviation is *P_SOP,W_* − *P_SOP,B_* = 0.00159. The maximum SOP deviation in dB is 10log_10_(0.003123/0.00159) = 3.09 dB.

**Figure 5:**
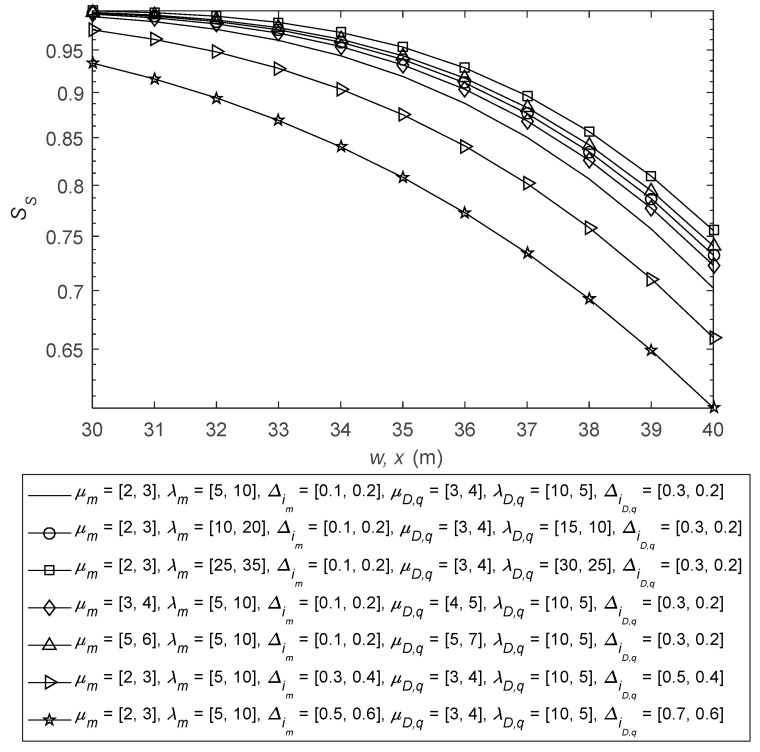
SSP analysis with varying D2D signals and MTW parameters at the relay and the receiver.

In Figure 5, SSP is given with varying MTW channel parameters of source-to-relay and relay-to-receiver D2D signals. Now, $r_{E1}$ = 15 cm, $r_{E1,l}$ = [9, 8] cm, $\mu_{R,k}$ = [4, 3], $\lambda_{R,k}$= [25, 20], $\Delta_{i_{R,k}}$ = [0.5, 0.4], $\mu_{D,u}$ = [3, 4], $\lambda_{D,u}$= [15, 20], $\Delta_{i_{D,u}}$ = [0.4, 0.6], $r_{E2}$ = 14 cm and $r_{E2,n}$ = [10, 8] cm. It can be observed from the figure that, by increasing the values of parameters $\mu_{m}$, $\lambda_{m}$, $\mu_{D,q}$ and $\lambda_{D,q}$, SSP performance improvement is observed due to enhanced SIR conditions. However, as the values of $\Delta_{i_{m}}$ and $\Delta_{i_{D,q}}$ are increased, degradation in SSP performance is seen due to detriment in SIR conditions.

**Figure 6:**
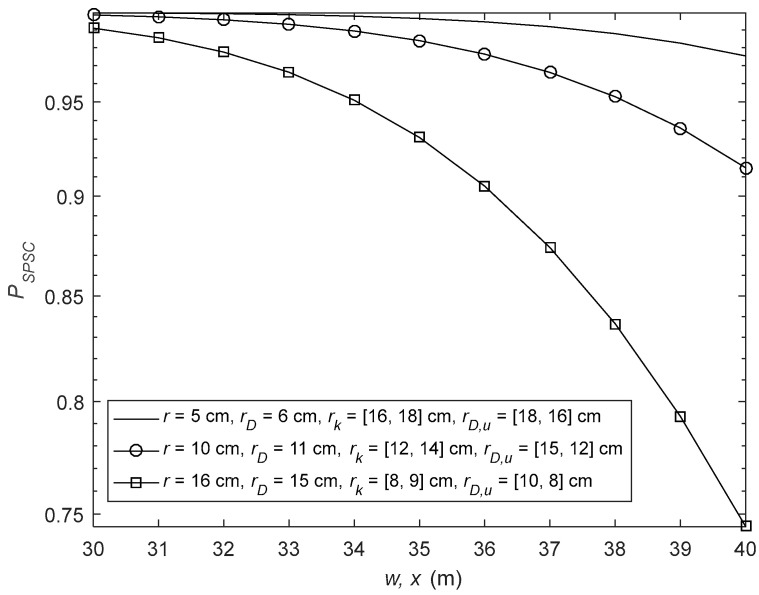
SPSC analysis for various pointing error values of the D2D signal and the CCI signal at the relay and the receiver.

In Figure 6, the SPSC analysis is given with different values of pointing errors of the D2D signal and the CCI signal at the relay and the receiver. The parameters are as follows: $r_{E1}$ = 8 cm, $r_{E1,l}$ = [18, 16] cm, $\lambda_{m}$= [10, 5], $\mu_{m}$ = [2, 3], $\Delta_{i_{m}}$ = [0.2, 0.3], $\mu_{R,k}$ = [4, 3], $\lambda_{R,k}$= [25, 20], $\Delta_{i_{R,k}}$ = [0.5, 0.4], $\mu_{D,q}$ = [3, 2], $\lambda_{D,q}$= [5, 10], $\Delta_{i_{D,q}}$ = [0.3, 0.2], $\mu_{D,u}$ = [3, 4], $\lambda_{D,u}$= [15, 20], $\Delta_{i_{D,u}}$ = [0.4, 0.6], $r_{E2}$ = 8 cm and $r_{E2,n}$ = [18, 16] cm. It can be seen that, when the pointing errors of the D2D signals are increased and the pointing errors of CCI are reduced at the relay and the receiver, the SPSC performance is degraded.

**Figure 7:**
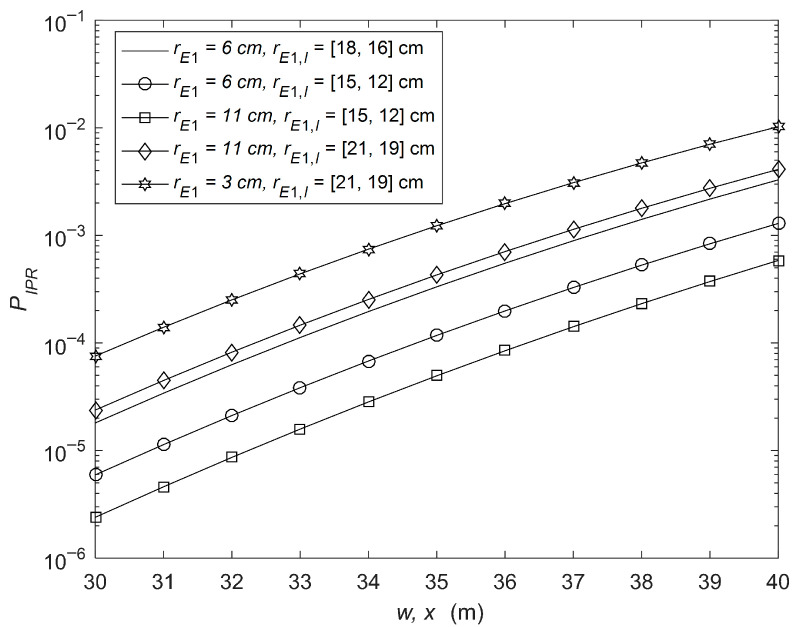
IP performance at the relay with varying pointing errors of the eavesdropper and eavesdropper’s CCI signals.

In Figure 7, the IP performance at the relay is given with varying pointing errors of the eavesdropper and eavesdropper’s CCI signals. The parameters are as follows: $\lambda_{m}$= [10, 5], $\mu_{m}$ = [2, 3], $\Delta_{i_{m}}$ = [0.2, 0.3], $\mu_{R,k}$ = [4, 3], $\lambda_{R,k}$= [25, 20], $\Delta_{i_{R,k}}$ = [0.5, 0.4], $\mu_{D,q}$ = [3, 2], $\lambda_{D,q}$= [5, 10], $\Delta_{i_{D,q}}$ = [0.3, 0.2], $\mu_{D,u}$ = [3, 4], $\lambda_{D,u}$= [15, 20], $\Delta_{i_{D,u}}$ = [0.4, 0.6], $r_{E2}$ = 8 cm and $r_{E2,n}$ = [18, 16] cm. From the figure, it is obvious that, by increasing the pointing errors of the eavesdropper, IP decreases. However, when the pointing errors of the eavesdropper are decreased, IP is increased. On the other hand, by increasing the pointing errors of eavesdropper’s CCI, IP is increased. And, when the pointing errors of eavesdropper’s CCI are decreased, IP is decreased.

**Figure 8:**
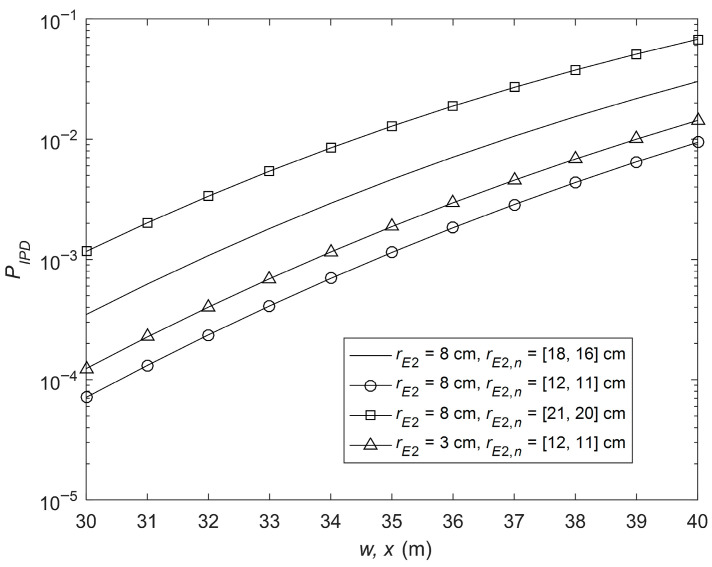
IP performance at the receiver with varying pointing errors of the eavesdropper and eavesdropper’s CCI signals.

In Figure 8, the IP performance at the receiver is given with varying pointing errors of the eavesdropper and eavesdropper’s CCI signals. The parameters are as follows: $\lambda_{m}$= [10, 5], $\mu_{m}$ = [2, 3], $\Delta_{i_{m}}$ = [0.2, 0.3], $\mu_{R,k}$ = [4, 3], $\lambda_{R,k}$= [25, 20], $\Delta_{i_{R,k}}$ = [0.5, 0.4], $\mu_{D,q}$ = [3, 2], $\lambda_{D,q}$= [5, 10], $\Delta_{i_{D,q}}$ = [0.3, 0.2], $\mu_{D,u}$ = [3, 4], $\lambda_{D,u}$= [15, 20], $\Delta_{i_{D,u}}$ = [0.4, 0.6], $r_{E1}$ = 7 cm and $r_{E1,l}$ = [15, 17] cm. Figure 8 shows a similar pattern to that of Figure 7.

**Figure 9:**
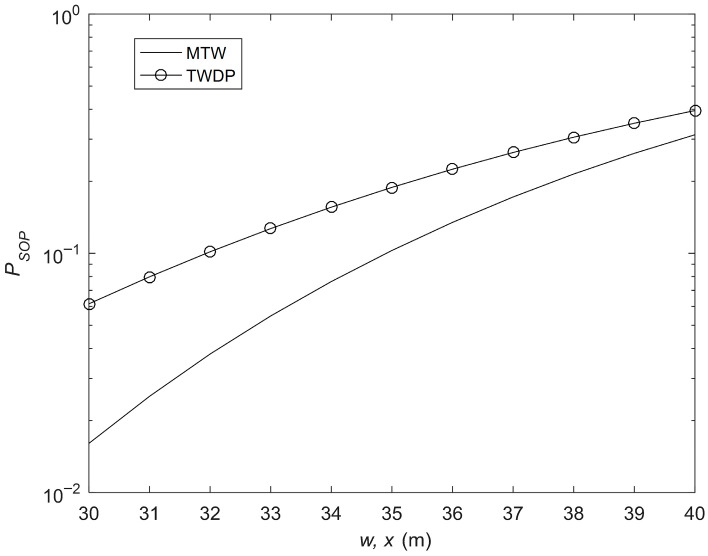
SOP performance comparison between the MTW and TWDP models.

Figure 9 illustrates the comparison of SOP performance between MTW and TWDP models. The TDWP model can be obtained from MTW by setting the number of clusters to 1 in MTW expressions [14]. The parameters are as follows: $r_{E1}$ = 15 cm, $r_{E1,l}$ = [9, 8] cm, $\mu_{m}$ = [3, 2], $\Delta_{i_{m}}$ = [0.4, 0.2], $\lambda_{m}$= [10, 20], $\mu_{R,k}$ = [4, 2], $\lambda_{R,k}$= [15, 10], $\Delta_{i_{R,k}}$ = [0.5, 0.4], $\mu_{D,q}$ = [4, 3], $\lambda_{D,q}$= [15, 10], $\Delta_{i_{D,q}}$ = [0.3, 0.5], $\mu_{D,u}$ = [2, 3], $\lambda_{D,u}$= [15, 10], $\Delta_{i_{D,u}}$ = [0.4, 0.6], $r_{E2}$ = 14 cm and $r_{E2,n}$ = [10, 8] cm. From the figure, it is clear that MTW shows better SOP performance than TWDP for the same channel conditions. It is because, as the number of clusters increases, it causes the receiver to combine energy from multiple independent clusters. It is usually unlikely for all clusters to experience deep fades simultaneously. This averaging effect smoothens out the severe fluctuations in the received power, leading to a more stable connection and a lower SOP.

## 4. Conclusions

This paper presents an analysis of the PLS for a DF D2D network operating over recently proposed MTW channels in the presence of THz propagation and CCI. Utilizing a CF-based approach, the authors derive mathematical expressions for SOP, SSP, SPSC, and IP, all of which are functions of diversity, interference, and fading parameters. To mitigate the effects of fading, MRC diversity schemes are employed at the relay and the receiver. Numerical results indicate that, while the implementation of diversity improves secrecy outage performance, an increase in the number of clusters containing specular components leads to higher secrecy outage. CCI MTW parameters showed negligible impact on secrecy outage. Furthermore, secrecy success performance improves with a higher number of D2D signal’s clusters and a higher ratio of the specular to diffuse power. However, secrecy success performance degrades as the value of the parameter measuring the asymmetry between specular components in a cluster increases. The probability of SPSC worsens with increased D2D pointing errors and reduced interference pointing errors. Future work will consider a signal-to-interference-plus-noise ratio (SINR)-based analysis, the effect of mobile eavesdroppers, and machine learning approaches for PLS over co-channel interference-limited THz channels.

## Figures and Tables

**Table 1:** Comparison of our key contributions with previous works.

Refs.	Channel	Interference	Diversity	Security Metrics	Analysis
[8]	Rayleigh	Multiple access interference (MAI) and jamming interference	Cooperative diversity (amplify-and-forward relaying)	Secrecy rate	Theoretical Mathematical Analysis
[9]	Line-of-Sight (LoS)	None	None	Secrecy rate	Mathematical optimization
[10]	Nakagami-*m*	None	RIS-Assisted Beamforming	Secrecy outage probability, average secrecy rate, intercept probability and probability of non-zero secrecy capacity	Analytical approximation
[11]	Mixture Gamma (MG)	None	None	Average secrecy rate and secrecy outage probability	Closed-form approximations and asymptotic analysis
[12]	FTR	None	None	Secrecy capacity, secrecy outage probability and the probability of strictly positive secrecy capacity	Exact analysis
[13]	M-Distributed	None	None	Secrecy outage probability, ergodic secrecy capacity and probability of strictly positive secrecy capacity	Lower-bound and closed-form expressions
This Work	MTW	CCI	MRC	Secrecy outage probability, secrecy success probability, probability of strictly positive secrecy capacity and intercept probability	CF-based analysis

**Table 2:** Nomenclature for Figure 1.

Description	Symbol
Source	▀
Receiver	⬟
Relay	⬤
Eavesdropper	▲
D2D source-to-relay signal	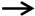
Relay-to-D2D receiver signal	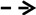
Relay/D2D source-to-eavesdropper signal	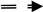
D2D source-to-relay distance	w
Relay-to-D2D receiver distance	x
D2D source-to-eavesdropper distance	y
Relay-to-eavesdropper distance	z
CCI source	◆
CCI signal	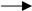
k−th CCI-to-relay distance	$p_{k}$
l−th CCI-to-eavesdropper (near relay) distance	$q_{l}$
u−th CCI-to-receiver distance	$r_{u}$
n−th CCI-to-eavesdropper (near receiver) distance	$s_{n}$

**Table 3:** Assumptions and fixed parameter values.

Assumptions	
$G_{TX,S}=G_{TX,k}=\cdots =G_{TX,K}=G_{TX,E1,l}=\cdots =G_{TX,E1,L},$ $G_{TX,R}=G_{TX,u}=\cdots =G_{TX,U}=G_{TX,E2,n}=\cdots =G_{TX,E2,N}$	
**Parameters**	**Values**
$P_{S}\;$	31.76 dBm
$P_{R}\;$	31.46 dBm
c	3 × 10^8^ m/s
f	300 GHz
a	10 cm
$C_{th}$	0.3 bits/s/Hz
$\omega_{x}$	60 cm
$\phi_{R}$	50%
$p_{R}$	101,325 Pa
$T_{R}$	296 °K
K	2
L	2
U	2
N	2
$P_{R,k}$	[28.45, 28.129] dBm
$P_{E1,l}$	[29.294, 29.031] dBm
$P_{D,u}$	[28.451, 28.129] dBm
$P_{E2,n}$	[29.294, 29.031] dBm
$p_{k}$	[40, 38] m
$r_{u}$	[33, 35] m
$q_{l}$	[30, 32] m
$s_{n}$	[30, 34] m
y	33 m (except Figure 3)
z	34 m (except Figure 3)
*r*	6 cm (except Figure 6)
$r_{k}$	[18, 16] cm (except Figure 6)
$r_{D}$	5 cm (except Figure 6)
$r_{D,u}$	[15, 16] cm (except Figure 6)

## Data Availability

Data is contained within the article.
